# Characterising the Economic Burden of Functional Dyspepsia: A Cost‐of‐Illness Study of Direct and Indirect Costs

**DOI:** 10.1002/ueg2.70257

**Published:** 2026-07-30

**Authors:** Daan H. C. A. Bosch, Brigitte A. B. Essers, Abraham B. Beckers, Johanna T. W. Snijkers, Bjorn Winkens, Ad A. M. Masclee, Daniel Keszthelyi

**Affiliations:** ^1^ Department of Gastroenterology and Hepatology Maastricht University Medical Centre+ Maastricht the Netherlands; ^2^ NUTRIM School of Nutrition and Translational Research in Metabolism Maastricht University Maastricht the Netherlands; ^3^ KEMTA Department of Clinical Epidemiology and Medical Technology Assessment Maastricht University Medical Centre+ Maastricht the Netherlands; ^4^ CAPHRI Care and Public Health Research Institute Maastricht University Maastricht the Netherlands; ^5^ Department of Methodology and Statistics Maastricht University Maastricht the Netherlands

**Keywords:** cost‐of‐illness, disorder of gut‐brain interaction, economic burden, functional dyspepsia, quality of life

## Abstract

**Background:**

Functional dyspepsia (FD) is a prevalent disorder of gut‐brain interaction (DGBI) and is associated with substantial impact on health care utilisation. However, analyses examining patient‐specific characteristics that contribute to this burden remain limited. This study assessed the economic burden of patients with FD and examined patient‐specific characteristics related to costs.

**Methods:**

Patients who had been recruited for a randomised controlled trial completed several questionnaires. Health care utilisation and productivity losses were measured using the iMTA Medical Consumption Questionnaire and Productivity Cost Questionnaire. Participants also completed the EuroQol 5‐Dimension 5‐Level, Nepean Dyspepsia Index, Generalised Anxiety Disorder‐7, and Patient Health Questionnaire‐9. Non‐parametric bootstrapping was applied to calculate the mean annual costs. Multivariable linear regression was used to identify patient‐specific characteristics associated with costs.

**Results:**

Data were obtained from 73 participants (median age 39 years; 75% female). Mean annual total costs accounted for €12,192 (2.5th to 97.5th percentiles, €1125 to €36,369), of which €10,240 (2.5th to 97.5th percentiles, €0 to €34,771) were indirect costs. Higher disease‐specific quality of life (QoL) was associated with lower annual total costs (regression slope = −€4726 per 0.1‐point increase in QoL; 95% CI, −€7636 to −€1815; *p* = 0.002), while symptom severity, generic health‐related QoL or scores for anxiety or depression were not significantly associated.

**Conclusion:**

Patients with poorer disease‐specific QoL, but not necessarily those with more severe symptoms or higher scores for anxiety and depression, are particularly likely to incur higher annual costs. Understanding the nature of costs and associated patient characteristics is essential for enabling more targeted strategies.

**Trial Registration:**

The TENDER trial was registered on Clinicaltrials.gov under NCT03652571 on August 17, 2018.

## Key Summary

- Summarise the established knowledge on this subject
  ◦ Functional dyspepsia (FD) is a disorder of gut brain interaction.
  ◦ Previous studies have shown that FD is accompanied by substantial economic burden, but analyses regarding which patient‐specific characteristics are associated with this burden are lacking.
- What are the significant and/or new findings of this study?
  ◦ The current study shows that the mean annual total costs of patients with FD accounted for €12,192, of which 84% were related to productivity losses. This shows the need for more work‐related factors in treatment programs for FD.
  ◦ Disease specific quality of life, but not symptom severity, was significantly associated with costs, underscoring the importance of overall patient burden and not merely symptom improvement as a treatment outcome.

## Graphical abstract

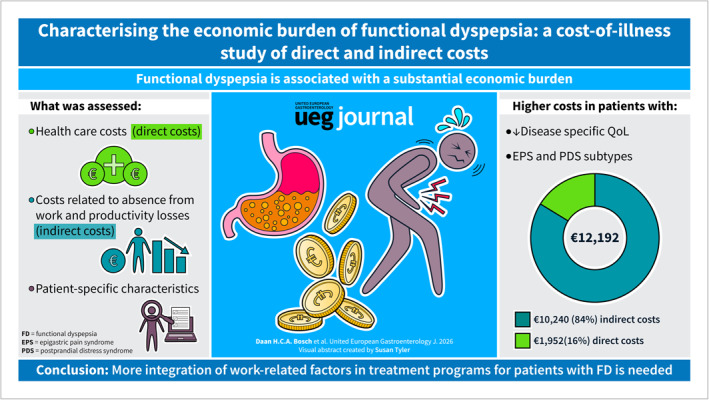

## 1 Introduction

Functional dyspepsia (FD) is a chronic condition defined as a complex of upper gastrointestinal (GI) symptoms including bothersome postprandial fullness, early satiation, epigastric pain and/or epigastric burning, without evidence of structural disease that is likely to explain the symptoms, based on the Rome V criteria [1]. FD can be subdivided into three subtypes: epigastric pain syndrome (EPS), postprandial distress syndrome (PDS) and overlap syndrome. The global prevalence is estimated to be 6.8%, with variations depending on geographic and demographic characteristics [2]. No biomarkers or disease‐specific symptoms are currently present to accurately distinguish FD from other causes of upper GI symptoms [3]. This makes it a challenging diagnostic process in clinical practice, often accompanied by the use of unnecessary procedures. Notably, more than 85% of upper GI endoscopies performed in patients with dyspepsia have normal findings [4]. Most treatment options currently recommended in guidelines are lacking evidence, making it an even more difficult condition to treat [5, 6]. Compared to other disorders of gut‐brain interaction (DGBIs), FD is one of the most prevalent conditions in secondary care and is associated with the highest health care burden [7]. The health care costs related to the treatment of FD per patient were $699 in 2009, according to an earlier report from the United States [8]. Moreover, FD has a substantial impact on physical, mental and social quality of life (QoL) [9]. Almost one third of patients with FD reported absenteeism and 77% reported reduced work productivity [10].

The substantial economic burden of FD may be attributable to the absence of diagnostic biomarkers and the limited evidence base supporting treatment options. A population‐based study reported that around 80% of patients with FD sought health care support in 12 months, with greater abdominal pain frequency being associated with more health care seeking for functional GI symptoms [11]. Moreover, FD is frequently accompanied by psychosocial comorbidities [12]. Increased levels of psychological distress, rather than the presence of a formal psychiatric diagnosis, seem to be associated with frequent health care seeking behaviour in patients with FD [13, 14].

Previous studies compared health care costs of employees with and without FD [15], or assessed work productivity and health care utilisation in patients diagnosed with FD without reporting costs [10, 16]. Other studies examining FD‐related costs were conducted decades ago [8, 17, 18, 19], suggesting a need for updated analyses. In addition, research on patient‐specific characteristics related to economic burden remains limited despite the annual increase in health care costs [20]. However, this may be relevant in facilitating more efficient and targeted care with the aim of reducing health care costs.

The current study aimed to examine the economic burden of FD by estimating costs which include both health care costs (direct costs) and costs related to productivity losses (indirect costs). In addition, this study includes the first in‐depth analysis to assess patient‐specific characteristics associated with costs among patients with FD. We hypothesised that more severe FD symptoms, higher anxiety and depression scores, and worse generic health‐related and disease‐specific QoL would be significantly associated with higher costs.

## 2 Materials and Methods

### 2.1 Study Design and Population

The analyses used baseline data from participants included in a multicentre, double‐blind, placebo‐controlled trial (TENDER trial) investigating the efficacy of nortriptyline in an escalating dose regimen, of which the primary results are reported elsewhere [21]. Patients with FD were recruited between 2018 and 2023 from the outpatient clinics of 11 Dutch hospitals. Furthermore, local advertisements were used to include patients with FD from primary care. Patients aged between 18 and 65 years who met the Rome IV criteria for FD were included [22]. Patients with evidence of current anxiety or depression (based on a score ≥ 10 on the Generalised Anxiety Disorder‐7 [GAD‐7] [23] and Patient Health Questionnaire‐9 [PHQ‐9] [24] and confirmed by a detailed interview by the investigator) were excluded from participation. Further details of the study protocol can be found in the Supporting Information S1 and at https://mdl.mumc.nl/sites/mdl/files/2024‐06/C1.%20TENDER%20study%20protocol%20version%202.1.pdf.

### 2.2 Data Collection

#### 2.2.1 Costs

A bottom‐up approach was used incorporating all health care costs, henceforth direct costs, and costs related to productivity losses, henceforth indirect costs, at an individual level that were likely related to the burden of FD. Data regarding health care utilisation and productivity losses were collected using the iMTA Medical Consumption Questionnaire (iMCQ) [25] and iMTA Productivity Cost Questionnaire (iPCQ) [26] respectively at the time of inclusion in the trial. The iMCQ is a validated self‐reported questionnaire to capture all medical resource use (e.g., outpatient and general practitioner visits, hospitalisations, paramedical care and medication use) over a 3‐month period. The iPCQ is a validated self‐reported questionnaire to assess health‐related productivity losses (including absenteeism, presenteeism and reduced productivity related to unpaid labour) over a period of 4 weeks. Both questionnaires are non‐disease‐specific, meaning that they are designed to capture resource use that is not solely related to a specific symptom or disorder. Respondents might experience difficulties in identifying disease‐related health care utilisation, as it may be unclear to them which symptoms are attributable to the disease. However, an extra question was added to the iMCQ to register specific visits to the general practitioner, the gastroenterology outpatient clinic, or emergency department that were likely due to symptoms of FD, as self‐reported by the participant. Costs related to physiotherapy, ergotherapy, speech therapy, acupuncture or appointments to the occupational health physician could not be excluded with complete certainty as being unrelated to symptoms of FD. Given that FD can co‐occur with other comorbid conditions, these therapeutic interventions may contribute, either directly or indirectly, to the improvement of symptoms of FD. Consequently, these associated costs were taken into account in the current study.

#### 2.2.2 Disease‐Specific and General Health Measures

Participants completed several validated questionnaires for the assessment of generic health‐related QoL using the EuroQol 5‐Dimension 5‐Level (EQ‐5D‐5L) [27] and disease‐specific QoL with the Nepean Dyspepsia Index (NDI) [28].

Anxiety and depression scores were assessed using the GAD‐7 and the PHQ‐9 respectively. The Rome IV for irritable bowel syndrome (IBS) [29] was completed by all participants.

All participants completed a generic questionnaire on among others smoking status and educational level.

All questionnaires (iMCQ, iPCQ, EQ‐5D‐5L, NDI, GAD‐7, PHQ‐9, Rome IV and generic questionnaire) were completed during the baseline measurement of the TENDER trial at the time of inclusion but before the run‐in period and randomisation.

Symptom severity was measured using a daily diary for 14 days containing five questions about the cardinal symptoms of FD, including epigastric pain, epigastric burning, upper abdominal bloating, postprandial fullness and early satiation (scale from zero [no symptoms] to ten [worst symptoms]).

### 2.3 Data Analyses

All cost calculations and analyses were performed following the international and Dutch guidelines (ISPOR—The Professional Society for Health Economics and Outcomes Research and the Dutch National Health care institute) [30, 31, 32]. All costs were extrapolated from 3 months (iMCQ) and 4 weeks (iPCQ) to 1 year. Cost prices from 2022 were adjusted to 2024 using the inflation factor derived from Centraal Bureau voor de Statistiek (Statistics Netherlands; https://www.cbs.nl/). Of note, long‐term absenteeism costs were adjusted by applying the friction method, which considers only the period needed to replace a worker and restore workplace productivity (which is maximised at 85 days).

### 2.4 Statistical Analyses

Statistical analyses were performed using IBM SPSS Statistics version 29.0.2.0 (20) and Microsoft Excel. Baseline characteristics were presented as mean (standard deviation [SD]) or median (interquartile range [IQR, 25th to 75th percentiles]) for numerical variables. Categorical variables were reported as absolute frequencies with corresponding percentages. To analyse differences in baseline characteristics between the FD subtypes, the Kruskal Wallis test, the Fisher's exact test, and Mann‐Whitney *U* test were used. Multivariable linear regression was performed to assess whether demographic, GI or psychological characteristics were associated with costs (model A: main model; model A’: main model with age, sex, smoking and educational level included; model B: main model with symptom severity of individual symptoms). A *p*‐value of ≤ 0.05 was considered statistically significant. Several post hoc sensitivity analyses were performed (model C: leaving out GAD‐7, PHQ‐9, symptom severity, EQ‐5D‐5L, NDI and comorbid IBS data; model D: entire model and indirect costs calculated without application of the friction method; and model E: leaving out GAD‐7, PHQ‐9, symptom severity, EQ‐5D‐5L, NDI and comorbid IBS data and indirect costs calculated without application of the friction method, Supporting Information S1: Methods).

Further information regarding data collection and analyses can be found in the supplementary material.

## 3 Results

### 3.1 Baseline Characteristics

In total, 73 participants (median age 39.0 years; 75% female) were included. Of these, 57 (78%) reported to be employed, while 3 (4%) were incapacitated for work, 5 (7%) were homemakers, and 2 (3%) reported to be retired. In addition, 16 (22%) participants were currently studying, some of whom were concurrently employed (Table 1). All participants were further divided according to the three FD subtypes (EPS [*n* = 19], PDS [*n* = 23] and overlap syndrome [*n* = 31]). Participants in the overlap syndrome had significantly higher symptom severity scores (compared to EPS: *p* < 0.001; compared to PDS: *p* = 0.031) and depression scores (compared to EPS: *p* = 0.084; compared to PDS: *p* = 0.002), and had significantly lower disease‐specific QoL (compared to EPS: *p* = 0.001; compared to PDS: *p* = 0.010) and generic health‐related QoL (compared to EPS: *p* = 0.200; compared to PDS: *p* = 0.004) compared to one or both of the other FD subtypes (Supporting Information S1: Table S1).

**TABLE 1:** Baseline characteristics.

	Total (*n* = 73)
Sex	
Female	55 (75%)
Male	18 (25%)
Age	
Years	39.0 (26.0–54.0)
(Previous) treatment setting	
Primary care	11 (15%)
Secondary care	45 (62%)
Tertiary care	11 (15%)
Combined secondary and tertiary care	6 (8%)
FD subtype (Rome IV)	
Epigastric pain syndrome	19 (26%)
Postprandial distress syndrome	23 (32%)
Overlap syndrome	31 (42%)
IBS (Rome IV)	
Yes	19 (26%)
IBS‐C	7 (10%)
IBS‐D	8 (11%)
IBS‐M	2 (3%)
IBS‐U	2 (3%)
Smoking status	
Never	45 (62%)
Current	8 (11%)
Former	20 (27%)
Educational level	
Lower	12 (16%)
Medium	33 (45%)
High	28 (38%)
Employment status	
Employed	57 (78%)^a^
Unemployed	0 (0%)
Incapacitated for work	3 (4%)
Homemaker	5 (7%)
Retired	2 (3%)
Currently studying	16 (22%)^a^
14‐day diary symptoms	
Composite total score symptom diary	4.38 (1.75)
Epigastric pain	4.71 (2.13)
Epigastric burning	3.52 (2.50)
Upper abdominal bloating	5.07 (2.60)
Postprandial fullness	5.27 (2.42)
Early satiation	3.33 (2.45)
NDI disease‐specific quality of life	
Total score	0.62 (0.22)
EQ‐5D‐5L generic health‐related quality of life	
Utility score	0.72 (0.21)
Psychological comorbidities	
Anxiety score (GAD‐7)	3.95 (3.37)
Depression score (PHQ‐9)	5.48 (4.04)

*Note:* Baseline characteristics were presented as mean (SD), median (IQR; 25th to 75th percentile), or *n* (%) from the entire study population (*n* = 73).

Abbreviations: EQ‐5D‐5L = EuroQol 5‐Dimension 5‐Level; FD = functional dyspepsia; GAD‐7 = Generalized Anxiety Disorder‐7; IBS = irritable bowel syndrome; IBS‐C = constipation‐predominant IBS; IBS‐D = diarrhoea‐predominant IBS; IBS‐M = mixed stool pattern IBS; IBS‐U = unspecified‐subtype IBS; NDI= Nepean Dyspepsia Index; PHQ‐9 = Patient Health Questionnaire‐9.

^a^ Some participants reported that they were both employed and concurrently studying.

### 3.2 Costs

**FIGURE 1:**
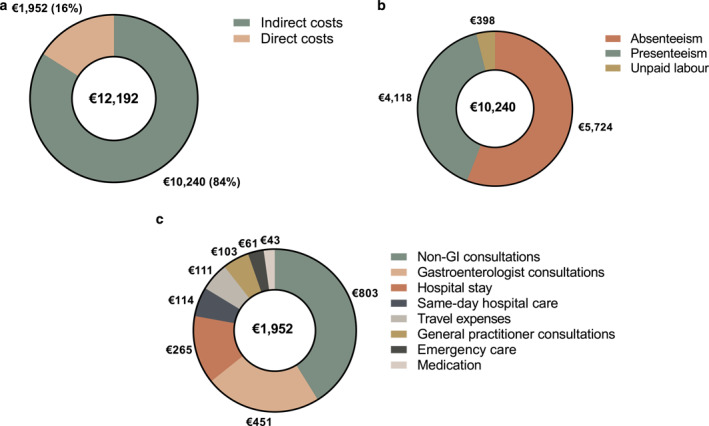
(a) Mean annual total costs split into direct and indirect costs for the entire study population (*n* = 73). (b) Mean annual indirect costs per category for the entire study population (*n* = 73). (c) Mean annual direct costs per category for the entire study population (*n* = 73).

Mean annual total costs were €12,192 (2.5th to 97.5th percentiles, €1125 to €36,369), of which €10,240 (84% of the total costs; 2.5th to 97.5th percentiles, €0 to €34,771) was attributable to indirect costs and €1952 (16% of the total costs; 2,5th to 97,5th percentiles, €271 to €6224) to direct costs (Figure 1a; Table 2).

**TABLE 2:** Societal annual costs.

Cost item	Unit price	Resource use per participant, Mean (SD)	Costs per participant, Mean (SD)	Bootstrapped costs per participant, Mean (2.5–97.5 percentile)
Total indirect costs			€10,240 (19,587)	€10,224 (0–34,771)^k^
Absenteeism	€42.75^a^	190.87 (486.02) hours	€5724 (15,696)	€5723 (2468–9628)
Presenteeism	€42.75^a^	96.31 (171.67) hours	€4118 (7339)	€4118 (2561–5917)
Unpaid labour	€20.15^a^	19.77 (74.81) hours	€398 (1508)	€401 (86–775)
Total direct costs			€1952 (2754)	€1976 (271–6224)^k^
Hospital stay	€690.37^a^	0.38 (3.28) days	€265 (2262)	€262 (0–794)
Medication	Various^b^ ^,^ ^c^		€43 (83)	€43 (26–63)
General practitioner appointment	€33.09^a^	3.12 (5.90) consultations	€103 (195)	€103 (63–151)
Gastroenterologist appointment	€128.64^a^	3.51 (4.76) consultations	€451 (612)	€452 (317–592)
Same‐day hospital care^j^	Various^d^ ^,^ ^e^	0.33 (1.29) procedures	€114 (425)	€115 (21–220)
Emergency and urgent care	€276.58^a^	0.11 (0.94) visits	€30 (259)	€31 (0–91)
Ambulance transportations	€566.02^a^	0.05 (0.47) transportations	€31 (265)	€31 (0–93)
Physiotherapist appointment	€41.79^a^	6.52 (25.65) contacts	€272 (569)	€271 (151–409)
Ergotherapy appointment	€26.07^a^	0.33 (2.81) contacts	€9 (73)	€9 (0–30)
Speech therapy appointment	€43.88^a^	0.66 (4.76) contacts	€29 (209)	€29 (0–82)
Acupuncture appointment	€60.00^b^ ^,^ ^f^	1.59 (4.70) contacts	€95 (282)	€96 (36–164)
Mental health care appointment	€161.46^b^ ^,^ ^g^	1.37 (4.23) contacts	€221 (683)	€221 (80–389)
Occupational health physician appointment	€120.00^b^ ^,^ ^h^	1.26 (3.46) consultations	€151 (415)	€151 (66–250)
Dietician appointment	€26.48^a^	0.99 (2.73) contacts	€26 (72)	€26 (12–44)
Travel expenses	€4.48^a^ ^,^ ^i^		€111 (120)	€111 (85–140)
Total costs			€12,192 (19,957)	€12,165 (1125–36,369)^k^

*Note:* Societal annual costs were presented for the entire study population (*n* = 73).

Abbreviation: SD = standard deviation.

^a^ Cost prices from 2022 were derived from the Dutch cost manual adjusted for inflation to 2024 prices (cost price of 2022 × 1.072 = cost price of 2024).

^b^ Cost prices from 2024.

^c^ Drug prices were derived from https://www.medicijnkosten.nl/ .

^d^ Cost prices from 2023 were derived from the finance department of the Maastricht University Medical Centre (cost price of 2023 × 1.033 = cost price of 2024).

^e^ Only day treatments and diagnostics related to gastrointestinal problems are included.

^f^ The cost price related to an appointment for acupuncture was estimated by listing several cost prices of different acupuncturists using Google.

^g^ The cost price related to a consultation of 60 min with a health psychologist, Section II (of the Dutch Individual Healthcare Professions Act—BIG) was derived from the Dutch Healthcare Authority (Nederlandse Zorgautoriteit, NZa).

^h^ The cost price related to a consultation of 30 min with an occupational health physician was estimated by asking a Dutch occupational health physician.

^i^ Travel expenses consist of a combination of the cost per kilometre driven (€0.28) and parking fees (€4.20).

^j^ Costs related to same‐day hospital care included performed gastroscopy (cost price 2024 €389.44; mean annual resource use per participant, 0.27 [SD, 1.02]) with annual mean costs per participant of (€107) and ultrasounds (cost price 2024 €126.77; mean annual resource use per participant, 0.05 [SD, 0.47] with annual mean costs per participant of €7).

^k^ As bootstrapping is applied, the sum of the individual cost items is thereby not equal to the total costs.

Absenteeism and presenteeism were the main drivers of indirect costs, accounting for €5724 (56% of the indirect costs; 2.5th to 97.5th percentiles, €2468 to €9628) and €4118 (40% of the indirect costs; 2.5th to 97.5th percentiles, €2561 to €5917), respectively (Figure 1b; Table 2). Of the total study population, 28% (*n* = 16) and 42% (*n* = 31) of participants reported illness‐related absenteeism and presenteeism, respectively, with mean annual hours of 190.87 (SD, 486.02) and 96.31 (SD, 171.67) (Table 2).

Direct costs were mainly driven by consultations with the general practitioner or gastroenterologist, with over half of the participants (56.2%) reporting to have consulted at least one of these health care professionals in response to symptoms likely related to FD. Participants had approximately three to four consultations on a yearly basis with their general practitioner (mean annual resource use per participant [SD], 3.12 [5.90]; mean annual costs €103 [2.5th to 97.5th percentiles, €63 to €151]) or gastroenterologist (mean annual resource use per participant [SD], 3.51 [4.76]; mean annual costs €451 [2.5th to 97.5th percentiles, €317 to €592]) likely related to FD symptoms (Figure 1c; Table 2). Procedures of same‐day hospital care (e.g., diagnostical procedures or outpatient treatments such as gastroscopy or ultrasound) showed a mean annual utilisation of 0.33 (SD, 1.29) per participant with corresponding mean annual costs of €114 (2.5th to 97.5th percentiles, €21 to €220) per participant.

### 3.3 Multivariable Linear Regression

Multivariable linear regression to assess patient characteristics associated with annual total costs showed that higher disease‐specific QoL was significantly associated with lower annual total costs (regression slope = −€4601 per 0.1 increase in disease‐specific QoL; 95% CI, −€7662 to −€1121; *p* = 0.013) (Table 3; model A). In addition, we found that the subtypes EPS (difference in means = €23,654; 95% CI, €9958 to €34,403; *p* = 0.017) and PDS (difference in means = €15,100; 95% CI, €5412 to €23,814; *p* = 0.025) were significantly associated with higher annual total costs compared to overlap syndrome (Table 3; model A). For direct costs, as well as generic health‐related QoL, symptom severity, and anxiety and depression scores, no significant associations were found (Model A). No significant associations could be found for each of the symptoms separately with annual total costs ([epigastric pain: regression slope = €1434; 95% CI, −€1370 to €3803; *p* = 0.338], [epigastric burning: regression slope = −€1563; 95% CI, −€3859 to €805; *p* = 0.186], [upper abdominal bloating: regression slope = −€315; 95% CI, −€3133 to €2624; *p* = 0.807], [postprandial fullness: regression slope = −€21; 95% CI, −€3030 to €2483; *p* = 0.988], [early satiation: regression slope = €612; 95% CI, −€1523 to €2962; *p* = 0.592]) (Supporting Information S1: Supporting Information 5 Table S3; model B). Multivariable linear regression with model C (removing symptom severity, disease‐specific QoL, generic QoL, comorbid IBS, and psychological comorbidities) showed no significant associations between FD subtypes and costs (Supporting Information S1: Supporting Information 6 Table S4; model C).

**TABLE 3:** Multivariable linear regression, model A.

	Difference in means/regression slope	95% CI	*p*‐value
Total costs			
EPS^a^	€23,654	€9958 to €34,403	**0.017**
PDS^a^	€15,100	€5412 to €23,814	**0.025**
Overlap syndrome^a^	€0		
Comorbid IBS^a^	€8391	€116 to €15,790	0.087
No comorbid IBS^a^	€0		
Depression score^b^	€1685	−€292 to €3852	0.196
Anxiety score^b^	–€982	−€2998 to €1059	0.348
Symptom severity^b^	–€395	−€4161 to €3261	0.841
Disease‐specific QoL^b^	–€4601	−€7662 to −€1121	**0.013**
Generic health‐related QoL^b^	€1417	−€1303 to €3733	0.337
Direct costs			
EPS^a^	€901	−€1065 to €2654	0.326
PDS^a^	–€21	−€1079 to €918	0.973
Overlap syndrome^a^	€0		
Comorbid IBS^a^	€1753	€231 to €3567	0.141
No comorbid IBS^a^	€0		
Depression score^b^	€128	−€138 to €429	0.484
Anxiety score^b^	€92	−€129 to €377	0.429
Symptom severity^b^	€29	−€603 to €460	0.908
Disease‐specific QoL^b^	–€268	−€649 to €88	0.248
Generic health‐related QoL^b^	€175	−€159 to €504	0.283
Indirect costs			
EPS^a^	€22,754	€8650 to €33,842	**0.025**
PDS^a^	€15,122	€6066 to €23,573	**0.023**
Overlap syndrome^a^	€0		
Comorbid IBS^a^	€6638	−€1464 to €14,023	0.171
No comorbid IBS^a^	€0		
Depression score^b^	€1557	−€474 to €3876	0.239
Anxiety score^b^	−€1074	−€3094 to €894	0.301
Symptom severity^b^	−€424	−€4197 to €3231	0.829
Disease‐specific QoL^b^	−€4334	−€7390 to −€826	**0.016**
Generic health‐related QoL^b^	€1241	−€1664 to €3723	0.422

*Note:* Multivariable linear regression with model A to analyse associations between total, direct and indirect costs, and patient characteristics in the entire study population (*n* = 73). Data were presented in difference of means or regression slope depending on the classification of the variable being either categorical (a) or numerical (b), with 95% CI intervals. Bold indicates *p*‐value of ≤ 0.05 was considered statistically significant. Model A included the following variables: FD subtype (epigastric pain syndrome [EPS]; postprandial fullness syndrome [PDS]; overlap syndrome); comorbid IBS (yes; no); depression scores (numerical); anxiety scores (numerical); symptom severity (numerical); disease‐specific QoL (numerical); generic health‐related QoL (numerical). Indirect costs were calculated with the application of the friction method.

Abbreviations: CI = confidence interval; EPS = epigastric pain syndrome; IBS = irritable bowel syndrome; PDS = postprandial fullness syndrome; QoL = quality of life.

The other results of the post hoc sensitivity analyses can be found in the Supporting Information S1 (Supporting Information 6. Results and Supporting Information 4–9 Tables S2–S7).

## 4 Discussion

This cost‐of‐illness study assessed direct and indirect costs in a Dutch population of adult patients with FD without the evidence of substantial psychological comorbidity who had been recruited to participate in a randomised controlled trial. The mean annual total costs were €12,192, consisting of 84% indirect costs and 16% direct costs. Absenteeism and presenteeism were the main drivers of the indirect costs, together accounting for 96% of these costs.

It is well‐established that FD is associated with increased health care utilisation and socioeconomic burden [7, 8, 9, 10, 12, 15, 16, 17, 18, 19, 33]. However, to our knowledge, this is the first study to actually examine the association between specific patient characteristics in relation to detailed analyses of both direct and indirect costs. The significant contribution of indirect costs to the total costs is in line with data of previous studies [10, 15, 16]. In our study, 28% of participants reported absence from work and 42% reported presenteeism. These findings highlight the substantial impact of FD on the daily functioning of patients. Given that indirect costs, attributable to absenteeism and presenteeism, contributed in a far greater degree to total costs compared to direct costs, work‐related aspects warrant greater integration into treatment programs for FD and other DGBIs [34]. Interestingly, mean annual total costs for FD were almost 17% higher compared to IBS, based on a recent study from our group using the same methodology, after correction for inflation (2020–2024, inflation factor of 1.21) [35]. The substantially higher costs of FD may be explained by the disease‐specific QoL, which appears to be strongly associated with costs. In the current study, the disease‐specific QoL of participants with FD was 0.11 points lower than that of the IBS population in the previous study of our group, which could explain this discrepancy.

Concordant with our hypothesis, we found that higher disease‐specific QoL was associated with lower total and indirect costs, which is also in line with our recent findings in IBS [35]. Interestingly, overall symptom severity as well as generic health‐related QoL, and anxiety and depression scores were not associated with costs in our study. In recent cost‐of‐illness studies in different DGBIs, an association with higher costs and more severe psychological burden was found, contrary to our results [35, 36]. Importantly, our study excluded patients with clear evidence of anxiety or depression. Therefore, the association between psychological burden and costs seen in our study may not be as predominant as in other studies [35, 36]. This could also explain the lack of association with generic health‐related QoL and costs, as generic health‐related QoL might rely largely on mental health, as we have recently shown in a study which also included participants from this study [37]. In addition, this observation also supports the notion that disease‐specific QoL can influence costs independently of psychological comorbidities.

Patients with overlap syndrome had significantly higher symptom severity and depression scores, and lower generic health‐related and disease‐specific QoL compared with those with EPS and/or PDS in the current study. A previous study has also shown that patients with the overlap syndrome have more severe symptoms, higher somatisation scores, and more frequent health care usage compared with patients with EPS or PDS [38]. Unexpectedly, we found the overlap syndrome to be significantly associated with lower costs compared with the EPS or PDS subtypes. Several post hoc sensitivity analyses were therefore conducted. First, we performed analyses with model C and found that the association between subtypes and costs was no longer statistically significant. This may show that the association between FD subtypes and costs is partially affected by symptom severity, QoL, and GI and psychological comorbidities. Second, we applied indirect costs without application of the friction method in models D and E and found that the subtype PDS was no longer significantly associated with higher costs compared with the overlap syndrome. Notably, none of the participants with EPS or PDS reported to be absent from work for more than 85 days, whereas three participants with overlap syndrome did. This shows that the application of the friction method can distort cost analyses by underestimating indirect costs in patients with a worse health state.

Strengths of this study include the comprehensive analysis of both direct costs and indirect costs in relation to patient characteristics. In addition, our study participants were included from primary, secondary and tertiary care settings, which enhances the generalisability to the overall FD population. Furthermore, we used questionnaires with a maximum recall period of 3 months to minimise recall bias.

Limitations of our study need to be mentioned. First, the relatively small sample size in relation to the large number of independent variables could have resulted in an underpowered model for models A, A′, B and D through overfitting. Additionally, analyses regarding FD subtypes, given the small sample, preclude us from drawing firm conclusions on the relative contributions of the different subtypes, in particular as the EPS subgroup is overrepresented in the current study as compared to available epidemiological data on FD [39]. For this reason, we have performed several sensitivity analyses to allow proper insight into the factors influencing the economic impact of FD. Second, our study consisted of patients who were willing to participate in a clinical trial investigating the efficacy of nortriptyline. Furthermore, patients with clear evidence of depression or anxiety were excluded from participation. In addition, patients with the use of psychotropic medication in the 3 months prior to the screening visit were excluded from participation as well. Psychological comorbidities, especially anxiety and depression, play an important role in FD [40] and can affect its economic impact. On the other hand, this might give a more accurate overview of direct costs that are less dependent on the presence of psychiatric comorbidity. We have also preselected participants based on their *CYP2D6* genotype before participation in the RCT, which could also have influenced costs, for instance those related to adverse events to certain medications. Therefore, a selection bias of participants may have occurred in this cost‐of‐illness study. Third, the data collected was self‐reported by the participants; therefore, social desirability bias cannot be entirely excluded. In addition, certain costs, such as those related to speech therapy and physiotherapy, might not be directly related to FD but rather to comorbidities often co‐occurring with FD. Fourth, although the analyses performed in this study demonstrated valuable associations, this does not mean that causality is demonstrated given the cross‐sectional nature of the study data. Fifth, participants could report gastroscopy as same‐day hospital care. However, there was no separate question included about diagnostic procedures, which likely resulted in an underestimation of these costs. Finally, the findings in this study might be specific for the Dutch health care and societal setting [41]. Still, the factors driving the economic burden found in this analysis are generic in nature and these results are applicable to many other health care systems.

In conclusion, FD imposes a substantial economic burden, with mean annual costs of €12,192, even in the absence of notable psychiatric comorbidity. These costs were primarily driven by productivity losses, accounting for more than 80% (€10,240) of the total costs. Higher disease‐specific QoL was significantly associated with lower costs, whereas the subtypes EPS and PDS were significantly associated with higher costs, although this could also be related to the cost analysis method applied. No significant associations could be found for costs and symptom severity, generic health‐related QoL, and psychological burden. These results suggest that the management of FD should not be merely focused on symptom reduction but more on the overall patient burden. Moreover, work‐related factors should be more systematically incorporated into FD treatment programs, given that productivity losses constitute the main driver of costs.

## Author Contributions

D.K. and A.A.M.M. contributed to the conceptualisation of the study. D.K. and A.A.M.M. acquired funding for the study. A.B.B. and D.K. contributed to the development of the methodology and protocol. B.W. and B.A.B.E provided input into the statistical analyses plan. D.H.C.A.B., A.B. and J.T.W.S. were responsible for data curation and project administration. D.H.C.A.B., A.B.B., J.T.W.S. and D.K. contributed to resources. D.K. supervised the study. D.H.C.A.B., B.A.B.E. and B.W. performed the formal analysis. D.H.C.A.B. wrote the original draft and was responsible for the visualization. B.A.B.E., A.B.B., J.T.W.S., B.W., A.A.M.M. and D.K. reviewed and edited the manuscript. D.H.C.A.B. and D.K. accessed and verified the data. The corresponding author attests that all listed authors meet the authorship criteria and that no other authors meeting the criteria have been omitted.

## Funding

The TENDER trial was funded by a grant received from ZonMw (the Netherlands Organisation for Health Research and Development), project number: 848016005.

## Ethics Statement

The study protocol of the TENDER trial was approved by the Maastricht University Medical Centre ethics committee on May 30, 2018 (protocol number NL62932.068.17) (applicable to all other participating centres) and the Boards of Directors of the respective hospitals.

## Consent

All patients gave informed consent before participation.

## Conflicts of Interest

D.K. received research funding from ZonMw, Rome Foundation, Horizon Europe, Horizon 2020, Dutch Foundation for Gastroenterology‐Hepatology (MDL Fonds) and speaker's fee from Rome Foundation (paid to host institute).

## Supporting information


Supporting Information S1

## Data Availability

The data that support the findings of this study are available from the corresponding author upon reasonable request. The de‐identified data will be deposited on Datahub (https://www.datahubmaastricht.nl) 3 months after publication.
