# Supplementary material for: Characterising the Economic Burden of Functional Dyspepsia: A Cost‐of‐Illness Study of Direct and Indirect Costs
Source: United European Gastroenterol J. 2026 Jul 30;14(7):e70257. doi: 10.1002/ueg2.70257 (PMC13421085; doi:10.1002/ueg2.70257)
Supplement: Supplementary file 1 — Supporting Information S1 [file UEG2-14-e70257-s001.docx]

**Supplementary material 1. Methods**

Study design and population
Patients were eligible for inclusion if they had insufficient effect on treatment with proton pump inhibitors. In the two years prior to inclusion, all patients had to have a negative stool or breath test for *Helicobacter Pylori* and upper gastrointestinal (GI) endoscopy was only mandatory in case of alarm features. Exclusion criteria were current use of psychotropic medication, history of gastric ulcer, liver or biliary disease, epilepsy, glaucoma, or previous major abdominal surgery or radiotherapy interfering with GI functioning.

Data collection
Disease-specific and general health measures
The EuroQol 5-Dimension 5-Level (EQ-5D-5L) consists of five domains (mobility, selfcare, daily activities, pain/discomfort, anxiety/depression). Participants were asked to give each domain a score on a scale from one (no problems) to five (extreme problems). The Nepean Dyspepsia Index (NDI) consists of four different domains: interference, knowledge/control, eat/drink, and sleep disturbance. Participants were asked to complete 25 questions regarding disease-specific quality of life (QoL) on a scale from one (not at all [best]) to five (severe [worst]), with a recall period of two weeks.
The Generalised Anxiety Disorder-7 (GAD-7) included seven questions, and the Patient Health Questionnaire-9 (PHQ-9) included nine questions on a scale from zero (not at all) to three (nearly every day). Both questionnaires have a recall period of two weeks.

Data analyses
Valuation of costs
Direct costs were calculated by multiplying the volume of resource use by the cost price per unit, derived from the Dutch costing manual (determined by the Dutch National Health care Institute)^1^ and from the Dutch Medicine cost website (official listings of drug prices: <https://www.medicijnkosten.nl/>). Costs regarding visits to outpatient clinics or same-day hospital care other than the gastroenterology and hepatology department were not included. Travel costs related to hospital visits, consultations by the general practitioner or paramedical care were calculated by multiplying the average kilometres patients travelled to each location with a standard price of €0.28, derived from the Dutch costing manual. An additional average parking fee of €4.20 was added per visit.
Indirect costs were calculated by multiplying the self-reported hours absent or inefficient working hours by average wage per hour, derived from the Dutch costing manual. For productivity losses in unpaid work, average wages for domestic help were used. Absenteeism and presenteeism were incurred for all employed participants, whereas productivity losses of unpaid work were incurred for all participants.

Quality of life and mental health
Utility scores from zero (worst generic health-related QoL) to one (best generic health-related QoL) were calculated by applying the Dutch EQ-5D-5L tariff to the health states derived from the participants’ EQ-5D-5L responses.^2^
For disease-specific QoL as measured with the NDI the incorporated scoring calculation of the validation study was used. First, participants’ answers to the questions were reversed so that a score of one reflects the worst QoL and a score of five reflects the best QoL. Second, questions were summed to compute four sub-scales: interference, knowledge/control, eat/drink, and sleep disturbance. Third, these sub-scores were adjusted to a zero (worst QoL) to 100 (best QoL) scale. A total disease-specific QoL score (on a scale from zero to 100) was obtained by summation of the scores of the four sub-scales and division by four. At last, this total disease-specific QoL score was divided by 100 to maintain a score on a scale from zero (worst QoL) to one (best QoL).
Questions from the GAD-7 or PHQ-9 were summed separately to obtain a total anxiety (scale zero [best] to 21 [worst]) or depression (scale zero [best] to 27 [worst]) score respectively.

Statistical analyses
Multivariable linear regression was performed to assess whether demographic, GI or psychological characteristics were associated with costs. The model included the following variables: functional dyspepsia (FD) subtype (epigastric pain syndrome [EPS]; postprandial distress syndrome [PDS]; overlap syndrome); comorbid irritable bowel syndrome (IBS) (yes; no); symptom severity (numerical); anxiety scores (numerical); depression scores (numerical); generic health-related QoL (numerical); disease-specific QoL (numerical) (model A). Separate analyses were performed for total, direct and indirect costs. To investigate whether age (years [numerical]), sex (male; female), smoking (current; former; never) and educational level (lower; medium; higher) could be significantly associated with costs, we included those variables to the model (model A’). We did not include age, sex, smoking and educational level in the other models in order to prevent overfitting of the data. We further evaluated whether the severity of each of the five symptoms of FD (epigastric pain, epigastric burning, upper abdominal bloating, postprandial fullness and early satiation [all continuous]) as per the 14-day diary score was individually associated costs by including these variables into the analyses for total, direct and indirect costs (model B). As cost data are typically highly skewed, non-parametric bootstrapping (with 10,000 replications) was used for calculation of 2.5^th^ and 97.5^th^ percentiles and for multivariable linear regression analyses, making the analyses less sensitive to violations of the normality assumption. Data was presented as difference in means for categorical variables and regression slope for numerical variables. Several post-hoc sensitivity analyses were conducted. Several post-hoc sensitivity analyses were conducted. First, a multivariable linear regression was performed to assess the association between FD subtypes and costs without including the variables symptom severity, anxiety and depression scores, generic health-related QoL, disease-specific QoL, and comorbid IBS (model C). Second, a multivariable linear regression was performed with indirect costs being calculated without application of the friction method, whereby costs were assigned to all days of absenteeism instead of only the maximum of 85 days (model D). Third, we performed a multivariable linear regression by using the calculation of indirect costs without applying the friction method, but with removal of symptom severity, anxiety and depression scores, generic health-related QoL, disease-specific QoL, and comorbid IBS from the model (model E).

**Supplementary material 2. Results**

Post-hoc sensitivity analyses
Model A’ showed no significant associations for age, sex, education levels and smoking status with costs (*Supplementary table 2; model A’)*. When Model D (including indirect costs calculated without application of the friction method) was used for the multivariable linear regression, we found that EPS was significantly associated with higher total costs (difference in means = €18,967; 95% CI, €6,772 to €30,07§; p = 0.010) and higher indirect costs (differences in means = €18,066; 95% CI, €5,482 to €29,932; p = 0.014) compared to the overlap syndrome (*Supplementary table 5; model D*). A higher disease-specific QoL was significantly associated with lower total costs (regression slope = -€6,254 per 0.1-point increase in disease-specific QoL; 95% CI, -€10,057 to -€1,350; p = 0.004) and with lower indirect costs (regression slope = -€5,987 per 0.1-point increase in disease-specific QoL; 95% CI, -€9,888 to -€1,129; p = 0.005) (*Supplementary table 5; model D*). At last, we used Model E (removing symptom severity, disease-specific QoL, generic QoL, comorbid IBS, and psychological comorbidities, with indirect costs calculated without application of the friction method) in the multivariable linear regression, but no significant associations between patient characteristics and costs were found (*Supplementary table 6; model E*). There were three participants with the overlap syndrome subtype who reported to be absent for more than 85 days. Thereby, the mean indirect costs for the overlap syndrome increased from €5,134 [€8,330] to €13,739 [€26,311] (*Supplementary table 7*), when not applying the fiction method. There were no participants with EPS or PDS who reported an absenteeism of more than 85 days.

| **Supplementary material 3 table 1. Baseline characteristics per subtype** | | | | |
| --- | --- | --- | --- | --- |
|  | **EPS**  **(n = 19)** | **PDS**  **(n = 23)** | **Overlap syndrome**  **(n = 31)** | **p-value** |
| **Sex** |  |  |  | 0.619 |
| Female | 13 (68%) | 17 (74%) | 25 (81%) |  |
| Male | 6 (32%) | 6 (26%) | 6 (19%) |  |
| **Age** |  |  |  |  |
| Years | 35.0 (27.0- 52.5) | 41.0 (24.0-52.0) | 39.0 (26.5-57.5) | 0.913 |
| **IBS** |  |  |  | 0.301 |
| Yes | 3 (16%) | 5 (22%) | 20 (65%) |  |
| No | 16 (84%) | 18 (78%) | 11 (35%) |  |
| **Smoking status** |  |  |  | 0.742 |
| Never | 12 (63%) | 16 (70%) | 17 (55%) |  |
| Current | 2 (11%) | 1 (4%) | 5 16%) |  |
| Former | 5 (26%) | 6 (26%) | 9 (29%) |  |
| **Educational level** |  |  |  | 0.441 |
| Lower | 1 (5%) | 3 (13%) | 8 (26%) |  |
| Medium | 9 (47%) | 11 (48%) | 13 (42%) |  |
| High | 9 (47%) | 9 39%) | 10 (32%) |  |
| **14-day diary symptoms** |  |  |  |  |
| Composite total score diary | 3.23 (1.70)^$^ | 4,20 (1,53)^^^ | 5.21 (1.53) | <0.001 |
| **NDI disease-specific QoL** |  |  |  |  |
| Total score | 0.73 (0.21)^%^ | 0.65 (0.18)^!^ | 0.52 (0.21) | 0.001 |
| **EQ-5D-5L generic health-related QoL** |  |  |  |  |
| Utility score | 0.74 (0.19)^&^ | 0.81 (0.14)^*^ | 0.64 (0.24) | 0.014 |
| **Psychological comorbidities** |  |  |  |  |
| Anxiety score (GAD-7) | 3.74 (3.75) | 3.70 (2.91) | 4.26 (3.51) | 0.704 |
| Depression score (PHQ-9) | 5.21 (4.22)^#^ | 3.65 (2.44)^@^ | 7.00 (4.36) | 0.008 |

Baseline characteristics were presented as mean (SD), median (IQR; 25^th^ to 75^th^ percentile), or n (%) from the entire study population (n = 73) per FD subtype. EPS= epigastric pain syndrome. PDS= postprandial fullness syndrome. IBS= irritable bowel syndrome. NDI= Nepean Dyspepsia Index. EQ-5D-5L= EuroQol 5-Dimension-5-Level. QoL= quality of life. GAD-7= Generalised Anxiety Disorder-7. PHQ-9= Patient Health Questionnaire-9.

$ Symptom severity score was significantly lower in participants with EPS compared to participants with overlap syndrome (p < 0.001) and compared to participants with PDS (p = 0.047).
^ Symptom severity score was significantly lower in participants with PDS compared to participants with overlap syndrome (p = 0.031).
% Disease-specific QoL was significantly higher in participants with EPS compared to participants with overlap syndrome (p = 0.001), but not compared to participants with PDS (p = 0.088).
! Disease-specific QoL was significantly higher in participants with PDS compared to participants with overlap syndrome (p = 0.010).
& Generic health-related QoL was not significantly higher in participants with EPS compared to participants with overlap syndrome (p = 0.200), but not significantly lower compared to participants with PDS (p = 0.131).
* Generic health-related QoL was significantly higher in participants with PDS compared to participants with overlap syndrome (p = 0.004).
# Depression score was not significantly lower in participants with EPS compared to participants with overlap syndrome (p = 0.084) and not significantly higher compared to participants with PDS (p = 0.531).
@ Depression score was significantly lower in participants with PDS compared to participants with overlap syndrome (p = 0.002).

| **Supplementary material 4 table 2. Multivariable linear regression, model A’** | | | |
| --- | --- | --- | --- |
|  | **Difference in means/ regression slope** | **95% CI** | **p-value** |
| **Total costs** |  |  |  |
| Age^$^ | -€49 | -€263 to €352 | 0.715 |
| Female^#^ | €2,494 | -€8,480 to €12,782 | 0.672 |
| Male^#^ | €0 |  |  |
| **EPS^#^** | €**23,079** | €**8,194 to** €**35,837** | **0.021** |
| **PDS^#^** | €**15,121** | €**5,915 to** €**24,363** | **0.018** |
| Overlap syndrome^#^ | €0 |  |  |
| Comorbid IBS^#^ | €8,816 | -€221 to €17,228 | 0.115 |
| No comorbid IBS^#^ | €0 |  |  |
| Education lower^#^ | -€11,189 | -€27,168 to €1,499 | 0.097 |
| Education medium^#^ | €236 | -€7,660 to €8,873 | 0.956 |
| Education higher^#^ | €0 |  |  |
| Smoking former^#^ | €5,437 | -€4,569 to €15,138 | 0.345 |
| Smoking current^#^ | -€1,412 | -€14,901 to €10,308 | 0.816 |
| Smoking never^#^ | €0 |  |  |
| Depression score^$^ | €1,550 | -€417 to €3,523 | 0.199 |
| Anxiety score^$^ | -€1,152 | -€3,214 to €977 | 0.271 |
| Symptom severity^$^ | €56 | -€3,482 to €3,645 | 0.974 |
| **Disease-specific QoL^$^** | **-**€**4,552** | **-**€**7,458 to -**€**1,321** | **0.011** |
| Generic health-related QoL^$^ | €649 | -€2,489 to €3,365 | 0.678 |
|  |  |  |  |
| **Direct costs** |  |  |  |
| Age^$^ | €29 | -€6 to €66 | 0.164 |
| Female^#^ | -€129 | -€1,414 to €1,152 | 0.834 |
| Male^#^ | €0 |  |  |
| EPS^#^ | €625 | -€1,628 to €2,503 | 0.550 |
| PDS^#^ | -€66 | €1,294 to €1,077 | 0.913 |
| Overlap syndrome^#^ | €0 |  |  |
| Comorbid IBS^#^ | €1,739 | €99 to €3,806 | 0.165 |
| No comorbid IBS^#^ | €0 |  |  |
| Education lower^#^ | -€994 | -€3,355 to €1,283 | 0.362 |
| Education medium^#^ | -€474 | -€1,696 to €852 | 0.528 |
| Education higher^#^ | €0 |  |  |
| Smoking former^#^ | -€938 | -€2,863 to €321 | 0.267 |
| Smoking current^#^ | -€1,143 | -€3,135 to €577 | 0.263 |
| Smoking never^#^ | €0 |  |  |
| Depression score^$^ | €189 | -€148 to €650 | 0.417 |
| Anxiety score^$^ | €57 | -€169 to €325 | 0.655 |
| Symptom severity^$^ | -€53 | -€646 to €359 | 0.833 |
| Disease-specific QoL^$^ | -€268 | -€711 to €207 | 0.257 |
| Generic health-related QoL^$^ | €130 | -€280 to €561 | 0.484 |
|  |  |  |  |
| **Indirect costs** |  |  |  |
| Age^$^ | €20 | -€293 to €338 | 0.877 |
| Female^#^ | €2,622 | -€8,138 to €12,232 | 0.657 |
| Male^#^ | €0 |  |  |
| **EPS^#^** | €**22,454** | €**7,387 to** €**35,462** | **0.031** |
| **PDS^#^** | €**15,187** | €**6,065 to** €**24,101** | **0.024** |
| Overlap syndrome^#^ | €0 |  |  |
| Comorbid IBS^#^ | €7,077 | -€1,872 to €15,366 | 0.194 |
| No comorbid IBS^#^ | €0 |  |  |
| Education lower^#^ | -€10,195 | -€24,645 to €1,221 | 0.116 |
| Education medium^#^ | €710 | -€7,817 to €9,871 | 0.876 |
| Education higher^#^ | €0 |  |  |
| Smoking former^#^ | €6,375 | -€3,620 to €16,382 | 0.284 |
| Smoking current^#^ | -€269 | -€16,211 to €13,463 | 0.963 |
| Smoking never^#^ | €0 |  |  |
| Depression score^$^ | €1,362 | -€632 to €3,356 | 0.264 |
| Anxiety score^$^ | -€1,209 | -€3,239 to €891 | 0.241 |
| Symptom severity^$^ | €109 | -€3,471 to €3,765 | 0.954 |
| **Disease-specific QoL^$^** | **-**€**4,284** | **-**€**7,218 to -**€**1,149** | **0.014** |
| Generic health-related QoL^$^ | €519 | -€2,607 to €3,219 | 0.758 |

Multivariable linear regression with model A’ to analyse associations between total, direct and indirect costs, and patient characteristics in the entire study population (n = 73). Data were presented in difference of means or regression slope depending on the classification of the variable being either categorical (#) or numerical ($), with 95% CI intervals. A p-value of ≤0.05 was considered statistically significant. CI= confidence interval. EPS= epigastric pain syndrome. PDS= postprandial fullness syndrome. IBS= irritable bowel syndrome. QoL= quality of life.

Model A’ included the following variables: age (years [numerical]); sex (male; female); FD subtype (epigastric pain syndrome [EPS]; postprandial fullness syndrome [PDS]; overlap syndrome); comorbid IBS (yes; no); education level (lower; medium; higher); smoking (current; former; never); depression score (numerical); anxiety score (numerical); symptom severity (numerical); disease-specific QoL (numerical); generic health-related QoL (numerical). Indirect costs were calculated with the application of the friction method.

| **Supplementary material 5 table 3. Multivariable linear regression, Model B** | | | |
| --- | --- | --- | --- |
|  | **Difference in means/ regression slope** | **95% CI** | **p-value** |
| **Total costs** |  |  |  |
| **EPS^#^** | **€23,436** | **€9,874 to €34,499** | **0.013** |
| **PDS^#^** | **€14,849** | **€4,507 to €24,645** | **0.037** |
| Overlap syndrome^#^ | €0 | NA | NA |
| Comorbid IBS^#^ | €7,184 | -€2,909 to €17,154 | 0.185 |
| No comorbid IBS^#^ | €0 | NA | NA |
| Depression score^$^ | €1,509 | -€454 to €3,616 | 0.202 |
| Anxiety score^$^ | -€804 | -€2,634 to €1,067 | 0.401 |
| Epigastric pain^$^ | €1,434 | -€1,370 to €3,803 | 0.338 |
| Epigastric burning^$^ | -€1,563 | -€3,859 to €805 | 0.186 |
| Upper abdominal bloating^$^ | -€315 | -€3,133 to €2,624 | 0.807 |
| Postprandial fullness^$^ | -€21 | -€3,030 to €2,483 | 0.988 |
| Early satiation^$^ | €612 | -€1,523 to €2,962 | 0.592 |
| **Disease-specific QoL^$^** | **-€4,325** | **-€7,504 to -€150** | **0.026** |
| Generic health-related QoL^$^ | €1,370 | -€1,584 to €4,037 | 0.368 |
|  |  |  |  |
| **Total direct costs** |  |  |  |
| EPS^#^ | €1,404 | -€417 to €3,557 | 0.183 |
| PDS^#^ | -€638 | -€2,155 to €662 | 0.353 |
| Overlap syndrome^#^ | €0 | NA | NA |
| Comorbid IBS^#^ | €1,523 | €19 to €3,337 | 0.159 |
| No comorbid IBS^#^ | €0 | NA | NA |
| Depression score^$^ | €85 | -€181 to €394 | 0.633 |
| Anxiety score^$^ | €79 | -€141 to €345 | 0.528 |
| Epigastric pain^$^ | -€71 | -€404 to €205 | 0.662 |
| Epigastric burning^$^ | -€288 | -€744 to €49 | 0.257 |
| Upper abdominal bloating^$^ | €210 | -€230 to €686 | 0.271 |
| Postprandial fullness^$^ | €149 | -€267 to €579 | 0.568 |
| Early satiation^$^ | -€96 | -€395 to €187 | 0.520 |
| Disease-specific QoL^$^ | -€391 | -€810 to €7 | 0.123 |
| Generic health-related QoL^$^ | €188 | -€146 to €504 | 0.269 |
|  |  |  |  |
| **Total indirect costs** |  |  |  |
| **EPS^#^** | **€22,032** | **€7,681 to €32,875** | **0.028** |
| **PDS^#^** | **€15,487** | **€5,230 to €24,578** | **0.040** |
| Overlap syndrome^#^ | €0 | NA | NA |
| Comorbid IBS^#^ | €5,661 | -€4,637 to €16,052 | 0.296 |
| No comorbid IBS^#^ | €0 | NA | NA |
| Depression score^$^ | €1,424 | -€542 to €3,434 | 0.234 |
| Anxiety score^$^ | -€883 | -€2,685 to €996 | 0.351 |
| Epigastric pain^$^ | €1,505 | -€1,423 to €4,015 | 0.316 |
| Epigastric burning^$^ | -€1,275 | -€3,642 to €1,056 | 0.286 |
| Upper abdominal bloating^$^ | -€525 | -€3,578 to €2,502 | 0.694 |
| Postprandial fullness^$^ | -€170 | -€3,185 to €2,490 | 0.907 |
| Early satiation^$^ | €709 | -€1,315 to €3,106 | 0.537 |
| **Disease-specific QoL^$^** | **-€3,933** | **-€7,061 to €52** | **0.039** |
| Generic health-related QoL^$^ | €1,182 | -€1,797 to €3,963 | 0.468 |

Multivariable linear regression with model B to analyse associations between total, direct and indirect costs, and patient characteristics in the entire study population (n = 73). Data were presented in difference of means or regression slope depending on the classification of the variable being either categorical (#) or numerical ($), with 95% CI intervals. A p-value of ≤0.05 was considered statistically significant. CI= confidence interval. EPS= epigastric pain syndrome. PDS= postprandial fullness syndrome. IBS= irritable bowel syndrome. QoL= quality of life.

Model B included the following variables: FD subtype (epigastric pain syndrome [EPS]; postprandial fullness syndrome [PDS]; overlap syndrome); comorbid IBS (yes; no); depression scores (numerical); anxiety scores (numerical); symptom severity of each of the five symptoms of FD (epigastric pain, epigastric burning, upper abdominal bloating, postprandial fullness and early satiation [all numerical]); disease-specific QoL (numerical); generic health-related QoL (numerical). Indirect costs were calculated with the application of the friction method.

| **Supplementary material 6 table 4. Multivariable linear regression, Model C** | | | |
| --- | --- | --- | --- |
|  | **Difference in means/ regression slope** | **95% CI** | **p-value** |
| **Total costs** |  |  |  |
| EPS^#^ | €11,742 | -€906 to €26,690 | 0.132 |
| PDS^#^ | €5,525 | -€1,916 to €14,002 | 0.209 |
| Overlap syndrome^#^ | €0 | NA | NA |
|  |  |  |  |
| **Direct costs** |  |  |  |
| EPS^#^ | -€183 | -€1,934 to €1,245 | 0.819 |
| PDS^#^ | -€829 | -€2,555 to €586 | 0.309 |
| Overlap syndrome^#^ | €0 | NA | NA |
|  |  |  |  |
| **Indirect costs** |  |  |  |
| EPS^#^ | €11,925 | -€676 to €26,848 | 0.123 |
| PDS^#^ | €6,354 | -€685 to €14,288 | 0.129 |
| Overlap syndrome^#^ | €0 | NA | NA |

Multivariable linear regression with model C to analyse associations between total, direct and indirect costs, and patient characteristics in the entire study population (n = 73). Data were presented in difference of means or regression slope depending on the classification of the variable being either categorical (#) or numerical ($), with 95% CI intervals. A p-value of ≤0.05 was considered statistically significant. CI= confidence interval. EPS= epigastric pain syndrome. PDS= postprandial fullness syndrome.

Model C included the following variables: FD subtype (epigastric pain syndrome [EPS]; postprandial fullness syndrome [PDS]; overlap syndrome). Indirect costs were calculated with the application of the friction method.

| **Supplementary material 7 table 5. Multivariable linear regression, Model D** | | | |
| --- | --- | --- | --- |
|  | **Difference in means/ regression slope** | **95% CI** | **p-value** |
| **Total costs** |  |  |  |
| **EPS^#^** | **€18,967** | **€6,772 to €30,071** | **0.010** |
| PDS^#^ | €9,660 | -€2,062 to €21,611 | 0.128 |
| Overlap syndrome^#^ | €0 | NA | NA |
| Comorbid IBS^#^ | €2,349 | -€8,188 to €11,948 | 0.641 |
| No comorbid IBS^#^ | €0 | NA | NA |
| Depression score^$^ | €532 | -€1,945 to €3,485 | 0.717 |
| Anxiety score^$^ | €1,539 | -€788 to €3,480 | 0.230 |
| Symptom severity | -€503 | -€4,782 to €4,059 | 0.816 |
| **Disease-specific QoL^$^** | **-€6,254** | **-€10,057 to -€1,350** | **0.004** |
| Generic health-related QoL^$^ | -€1,112 | -€4,689 to €2,263 | 0.534 |
|  |  |  |  |
| **Total direct costs** |  |  |  |
| EPS^#^ | €901 | -€1,065 to €2,654 | 0.326 |
| PDS^#^ | -€21 | -€1,079 to €918 | 0.973 |
| Overlap syndrome^#^ | €0 | NA | NA |
| Comorbid IBS^#^ | €1,753 | €231 to €3,567 | 0.141 |
| No comorbid IBS^#^ | €0 | NA | NA |
| Depression score^$^ | €128 | -€138 to €429 | 0.484 |
| Anxiety score^$^ | €92 | -€129 to €377 | 0.429 |
| Symptom severity | €29 | -€603 to €460 | 0.908 |
| Disease-specific QoL^$^ | -€268 | -€649 to €88 | 0.248 |
| Generic health-related QoL^$^ | €175 | -€159 to €504 | 0.283 |
|  |  |  |  |
| **Total indirect costs** |  |  |  |
| **EPS^#^** | **€18,066** | **€5,482 to €29,932** | **0.014** |
| PDS^#^ | €9,682 | -€1,735 to €21,335 | 0.118 |
| Overlap syndrome^#^ | 0 | NA | NA |
| Comorbid IBS^#^ | €596 | -€9,793 to €10,261 | 0.907 |
| No comorbid IBS^#^ | 0 | NA | NA |
| Depression score^$^ | €404 | -€2,186 to €3,478 | 0.782 |
| Anxiety score^$^ | €1,447 | -€1,028 to €3,446 | 0.266 |
| Symptom severity | -€532 | -€4,648 to €3,908 | 0.803 |
| **Disease-specific QoL^$^** | **-€5,987** | **-€9,888 to -€1,129** | **0.005** |
| Generic health-related QoL^$^ | -€1,287 | -€4,842 to €2,010 | 0.468 |

Multivariable linear regression with model D to analyse associations between total, direct and indirect costs, and patient characteristics in the entire study population (n = 73). Data were presented in difference of means or regression slope depending on the classification of the variable being either categorical (#) or numerical ($), with 95% CI intervals. A p-value of ≤0.05 was considered statistically significant. CI= confidence interval. EPS= epigastric pain syndrome. PDS= postprandial fullness syndrome. IBS= irritable bowel syndrome. QoL= quality of life.

Model D included the following variables: FD subtype (epigastric pain syndrome [EPS]; postprandial fullness syndrome [PDS]; overlap syndrome); comorbid IBS (yes; no); depression scores (numerical); anxiety scores (numerical); symptom severity (numerical); disease-specific QoL (numerical); generic health-related QoL (numerical). Indirect costs were calculated without the application of the friction method.

| **Supplementary material 8 table 6. Multivariable linear regression, Model E** | | | |
| --- | --- | --- | --- |
|  | **Difference in means/ regression slope** | **95% CI** | **p-value** |
| **Total costs** |  |  |  |
| EPS^#^ | €3,137 | -€12,865 to €24,944 | 0.719 |
| PDS^#^ | -€3,081 | -€16,283 to €9,372 | 0.622 |
| Overlap syndrome^#^ | €0 | NA | NA |
|  |  |  |  |
| **Total direct costs** |  |  |  |
| EPS^#^ | -€183 | -€1,984 to €1,230 | 0.818 |
| PDS^#^ | -€829 | -€2,667 to €607 | 0.319 |
| Overlap syndrome^#^ | €0 | NA | NA |
|  |  |  |  |
| **Total indirect costs** |  |  |  |
| EPS^#^ | €3,320 | -€12,698 to €20,707 | 0.701 |
| PDS^#^ | -€2,252 | -€14,653 to €9,317 | 0.719 |
| Overlap syndrome^#^ | €0 | NA | NA |

Multivariable linear regression with model E to analyse associations between total, direct and indirect costs, and patient characteristics in the entire study population (n = 73). Data were presented in difference of means or regression slope depending on the classification of the variable being either categorical (#) or numerical ($), with 95% CI intervals. A p-value of ≤0.05 was considered statistically significant. CI= confidence interval. EPS= epigastric pain syndrome. PDS= postprandial fullness syndrome.

Model E included the following variables: FD subtype (epigastric pain syndrome [EPS]; postprandial fullness syndrome [PDS]; overlap syndrome). Indirect costs were calculated without the application of the friction method.

| **Supplementary material 9 table 7. Societal annual costs per FD subtype** | | | |
| --- | --- | --- | --- |
|  | **EPS**  **(n = 19)** | **PDS**  **(n = 23)** | **Overlap syndrome**  **(n = 31)** |
| **Costs with application of the friction method** |  |  |  |
| Total costs | €19,137 (€30,466) | €12,919 (€18,750) | €7,395 (€9,297) |
| Direct costs | €2,077 (€1,745) | €1,432 (€1,869) | €2,261 (€3,673) |
| Indirect costs | €17,059 (€30,633) | €11,487 (€17,739) | €5,134 (€8,330) |
| **Costs without application of the friction method** |  |  |  |
| Total costs | €19,137 (€30,466) | €12,919 (€18,750) | €16,000 (€26,720) |
| Direct costs | €2,077 (€1,745) | €1,432 (€1,869) | €2,261 (€3,673) |
| Indirect costs | €17,059 (€30,633) | €11,487 (€17,739) | €13,739 (€26,311) |

Societal annual costs were presented for the entire study population (n = 73) per FD subtype. EPS= epigastric pain syndrome. PDS= postprandial fullness syndrome.

**Reference list**

1. Hakkaart-van Roijen L, Peeters S, Kanters T, et al. Costing manual: Methods.

2. M MV, K MV, S MAAE, et al. Dutch Tariff for the Five-Level Version of EQ-5D. *Value Health* 2016; 19: 343-352. 2016/06/22. DOI: 10.1016/j.jval.2016.01.003.
